# Motor actions influence subsequent sensorimotor decisions

**DOI:** 10.1038/s41598-017-16299-0

**Published:** 2017-11-21

**Authors:** Anna-Antonia Pape, Nima Noury, Markus Siegel

**Affiliations:** 1 Centre for Integrative Neuroscience & MEG Center, Otfried-Müller-Str. 25, University of Tübingen, 72076 Tübingen, Germany; 2 IMPRS for Cognitive and Systems Neuroscience, Österbergstr. 3, 72072 Tübingen, Germany

## Abstract

Sensorimotor decisions are influenced by factors beyond the current sensory input, but little is known about the effect of preceding motor actions. Here, we show that choice-unrelated motor actions influence subsequent sensorimotor decisions. By instructing participants to perform choice-unrelated motor responses before visuomotor decisions, we could manipulate upcoming decisions in a directed fashion. Subjects tended not to repeat the instructed motor response. Our results show that simple motor behaviors can influence subsequent sensorimotor decision.

## Introduction

Choice formation in sensorimotor decision making is influenced by many factors beyond the current stimulus such as reward expectations^1^, neural noise at sensory stages^2,3^, or previous decisions^4–6^. However, little is known about the effect of preceding motor actions on sensorimotor decisions because, traditionally, the motor system is considered merely as an output stage of the decision process. In contrast, recent evidence shows that also previous choice-related motor responses can influence sensorimotor decision making. Participants avoid repeating the same button-press for successive visual decisions with weak sensory evidence^7^. While this effect demonstrates an impact of previous motor responses on the current decision making process, it is not clear if this influence is restricted to previous motor responses made to report preceding choices, i.e. if it operates between successive decisions, like e.g. the Gambler’s fallacy^8,9^, or if alternatively, this influence reflects a more general effect of previous motor actions on decision making that is independent of successive decisions^10^. To address this, we tested if simple instructed motor actions can influence subsequent sensorimotor decisions.

## Results

**Figure 1:**
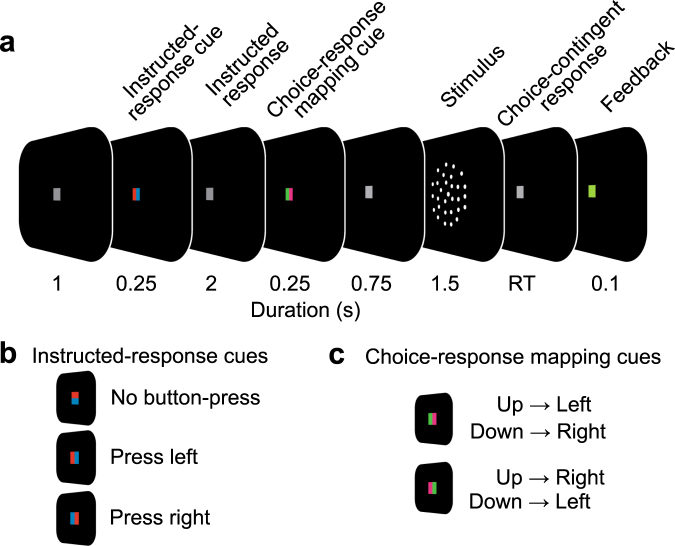
Visual-motion decision task and experimental manipulation (**a**) Participants reported the direction (up/down) of coherent motion in a display of randomly moving dots with a left- or right-hand button-press. For each trial, the mapping from choice to response was newly assigned with a cue before the stimulus (choice-response mapping cue). Furthermore, at the beginning of each trial, an instructed-response cue indicated subjects to either press the left button, the right button or no button. (**b**) Instructed-response cues. (**c**) Choice-response mapping cues.

Participants made decisions about the global motion direction (up/down) in patterns of randomly moving dots and reported their decision with a left or right hand button-press (Fig. 1a). The strength of global motion was adjusted for each participant to perform near the individual perceptual discrimination threshold (mean correct performance: 68.2 +/− 0.72% SEM). The mapping from choice (perceived motion direction: up/down) to response (pressed button: left/right) was randomly assigned on each trial and indicated by a choice-response cue shown before the stimulus (Fig. 1c). This design allowed us to dissociate the subjects’ choices (i.e. the reported percepts) from the subjects’ motor responses (i.e. movements made to report choices)^7,11^. To test if the influence of previous motor responses on subsequent decisions holds generally or is restricted to previous choice-related responses, we tested if we could manipulate the decision process by choice-unrelated motor actions. To this end, a color cue instructed participants to press either the left, right or no button before each perceptual decision (instructed response cue, Fig. 1b).

**Figure 2:**
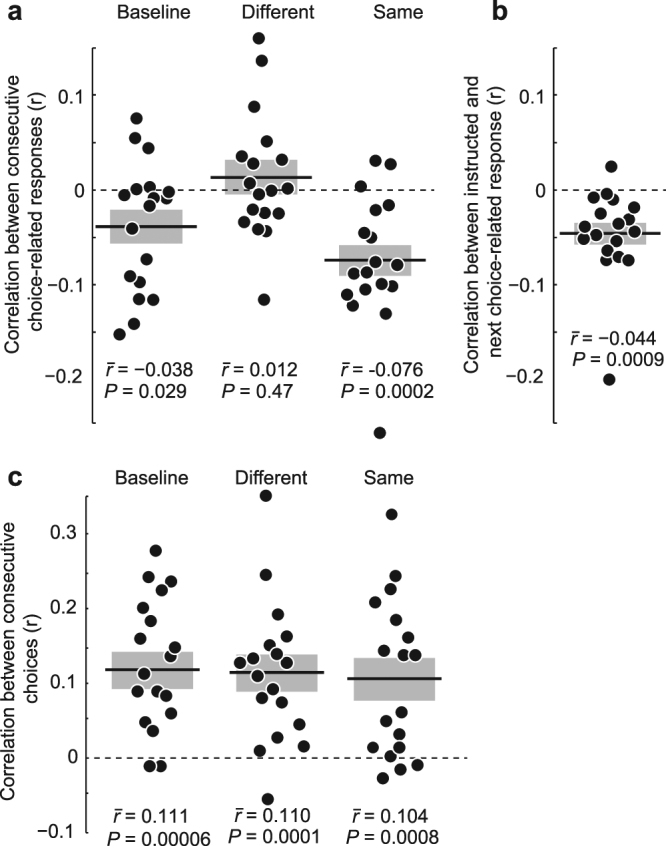
Effect of intermittent instructed button-presses. Each dot denotes one participant’s correlation-coefficient. Black lines and gray bars denote the mean and SEM of correlation coefficients across participants, respectively. For each panel, the group mean of correlation coefficients and the P-value of a t-test of Fisher-Z-transformed correlation coefficients against 0 are stated. (**a**) Correlation coefficients between consecutive choice-contingent button-presses. ‘Baseline’ condition: no intermittent button-press; ‘Different’ condition: instructed button-press different from preceding choice-response; ‘Same’ condition: instructed button-press is the same as preceding choice-response. (**b**) Correlation coefficients between instructed response and the following choice-contingent response. (**c**) Correlation coefficients between consecutive visual-motion decisions.

First, we focused on those consecutive trials, for which participants did not press a button in between two successive perceptual decisions (no button-press in Fig. 1b, baseline condition in Fig. 2a). We determined if there was an influence of the previous motor response with which subjects reported the preceding choice, on the current perceptual decision. We quantified this influence as a correlation between the side of successive choice-related button-presses. In accordance with previous results^7^, participants indeed showed a significant bias to alternate between motor responses when reporting consecutive visual-motion decisions (Fig. 2a, *P* = 0.029, mean *r*_Baseline_ = −0.038 +/− 0.06, *t*(17) = −2.38, *n* = 18, two-tailed one-sample t-test on Fisher-z-transformed correlation coefficients describing the relationship between choice-related responses in each participant). In other words, participants had a tendency to avoid pressing the same button twice^7^. In the following, we refer to this effect as ‘response- alternation bias’. This result confirms that motor responses made to report choices influence subsequent choices on a perceptual decision task^7^.

Next, we analyzed those consecutive trials for which participants pressed a button between two perceptual choices according to the instructed-response cue. If response alternation is driven by any previous motor act independent of whether it is made to report a choice or not, introducing instructed button-presses between perceptual decisions should interfere with the strength of response alternation observed in the baseline condition. Specifically, we hypothesized that instructing a button-press different from the previous choice-related button-press (Different-condition) should counteract, i.e. reduce, the response alternation between successive perceptual choices. Indeed, we found that an intermittent “different” button-press strongly reduced response alternation between two choice-related button-presses. (*r*_Baseline_ − *r*_Different_ = −0.050 +/− 0.10, *P* = 0.024, *t*(17) = −2.12, left-tailed paired t-test; *r*_Different_ = 0.012 +/− 0.01, *P = *0.47, *t*(17) = 0.74, two-tailed one-sample t-test, Fig. 2a). In other words, when participants pressed a button different to the previous choice-related button, this reduced their bias to avoid the previous choice-related button when reporting the next choice.

This result shows that instructed, choice-unrelated motor actions can manipulate response alternation between perceptual choices, suggesting that the impact of the instructed button-press reflects a general impact of motor actions on the following decision. Alternatively, the intermittent instructed button-press may simply have disturbed the response-alternation bias between choice-related button-presses. Introducing an instructed button-press repeating the previous choice-related button-press (Same-condition) can dissociate these two alternatives. If response alternation is motor-driven, the “same” button-press should leave response alternation untouched, or even strengthen it because the bias in favor of an alternation may build up over two button-presses. Otherwise, if response alternation is merely disturbed by introducing an intermittent button-press, a “same” intermittent button-press should induce the same reduction of response alternation as a “different” intermittent button-press.

As predicted by the first (motor-driven) hypothesis, with an intermittent “same” button-press, the tendency to avoid pressing the same key on the current choice-related response as on the previous choice-related response was significantly stronger than without any intermittent button-press (*r*_Baseline_ − *r*_Same_ = 0.038 +/− 0.08, *P* = 0.039, t(17) = 1.87, right-tailed paired t-test; *r*_*Same*_ = −0.076 +/− 0.07, *P* = 0.0002, t(17) = −4.6, two-tailed one-sample t-test, Fig. 2a).

In summary, we were able to selectively facilitate or counteract the tendency to alternate responses on subsequent decisions by instructing participants to press an intermittent button either repeating or alternating the previous choice-related response. This pattern of results suggests that motor actions in general bias responses on subsequent decisions towards the response that was not executed before. To directly test this, we next analyzed the relationship between the cued intermittent response and the following choice-related response. Indeed, we found that participants also had the tendency to alternate between the instructed button-press and the following decision (Fig. 2b, *r* = −0.044 +/− 0.011, *P* = 0.0009, *t*(17) = −3.99, two-tailed one-sample t-test). Furthermore, the strength of this effect depended on whether the cued response was the same or different to the previous choice-related response. There were more  response alternations from the instructed response to the next choice-response if the instructed response was a repetition of the previous choice-related button-press than when it was an alternation itself (*r*_Different_ − *r*_Same_ = −0.064 +/− 0.023, *P = *0.012, *t*(17) = 2.79, two-tailed paired t-test). In other words, the urge to avoid pressing the same button twice seemed to build up over two consecutive same button-presses, and therefore played out more strongly on the next decision. This interaction effect suggests a common neuronal substrate underlying response alternation for choice-related and instructed motor responses.

The fact that also choice-unrelated responses influence subsequent choices suggests that this response sequence effect acts on the response-selection stage rather than on the choice-selection or perceptual stage. In contrast, the bias to repeat the previous choice when faced with weak sensory evidence in successive visual decision-making tasks is thought to act on perceptual stages^4,6^. This sequence effect, often termed “choice-repetition bias”^5^, may reflect a bias of the visual system in adaptation to the constancy of visual inputs on short time scales. If choice repetition and response- alternation bias operate on different stages of the decision-making process, a choice-repetition bias should not be affected by intermittent instructed responses. Indeed, we found that subjects showed a significant tendency to repeat choices across subsequent visual-motion decisions (Fig. 2c, all conditions: *r* > 0.1, *P* < 0.001, *t*(17) > 4.0, two-tailed, one-sample t-tests, n = 18), but, as expected, this choice-repetition bias did not differ between baseline, same or different conditions (*r*_Baseline_−*r*_Same_: 0.0069 +/− 0.09, *P* = 0.739, *t*(17) = 0.338, two-tailed paired t-test; *r*_Baseline_ − *r*_Different_ = 0.0014, +/− 0.06, *P* = 0.918, *t*(17) = 0.10, two-tailed paired t-test).

## Discussion

Our results show that humans tend to alternate between motor responses when making perceptual decisions. Importantly, this response-alternation bias is independent of whether a previous motor response was made to report a previous perceptual decision or was a simple, instructed response. This suggests that previous motor acts in general have an impact on decision making and need to be considered in the design, analysis, and modeling of decision-making tasks.

The fact that also simple, choice-unrelated motor acts induce response alternation suggests that this bias operates on the response selection stage. This is in contrast to the choice repetition bias that likely involves perceptual stages^5,6^, or other abstract beliefs about sequences of independent events, such as the Gambler’s fallacy^8^.

The interpretation that response alternation acts on the response selection stage aligns well with a potential neural mechanism underlying the observed behavioral findings that is suggested by our previous research^7^. Response alternation may be driven by the neural aftermath of motor responses in motor cortex, i.e. the post-movement beta rebound^12^, which may inhibit response-repetition and bias response selection towards the previously not chosen response alternative^7^. While we did not record neural data to directly address this question here, our behavioral results are at least compatible with a motor-cortical beta-rebound driven mechanism. First, because response selection in visual decisions could be selectively biased not only by previous choice-related movement, but also by simple instructed movements in the same effector system. Second, because we found an interaction between previous choice-related and choice-unrelated motor responses, which suggests a common neuronal substrate that may well reside in motor cortex. Recordings of neural activity will be required to directly address this question.

We have tested our participants with index-finger button-presses. Therefore, an interesting open question concerns whether the observed response bias generalizes to other movements and effector systems. In fact, the response-alternation bias shown here bears strong similarities to a visual behavior known as “inhibition of return”, i.e. a delayed or reduced responding to previously attended visual targets that is known to be at least partly driven by motor processes^13,14^.

Our participants made visual decisions near perceptual threshold, i.e. they based their decisions on very weak sensory evidence. Further research is required to determine how the amount of sensory evidence shifts the reliance on visual input, the previous motor response and other factors influencing the visual decision, such as previous choices^5,6^, reward^15^ or motor effort^10,16^. Another interesting, related question concerns how the response-alternation bias plays out when participants are free to respond as soon as they want, i.e. in reaction-time tasks.

In summary, we show a response-alternation bias in perceptual decision making that is independent of the preceding percept or choice. Thus, simple instructed motor actions can manipulate subsequent decisions in the same effector system in a directed fashion.

## Methods

### Participants

18 healthy volunteers (11 female, mean age 28 years) participated in this study. All participants had normal or corrected-to-normal vision and received monetary reward for their participation. The study was conducted in accordance with the Declaration of Helsinki, and was approved by the ethics committee of the University of Tübingen. All participants gave written informed consent before participating.

### Behavioral Task

On each trial, participants decided if they saw coherent motion going upward or downward in a centrally presented dynamic random dot pattern and reported their percept (up/down) by button-press with the left or the right index finger (Fig. 1a, 2-alternative forced choice). The choice-response mapping was newly assigned on each trial by a color cue (Fig. 1c) whose orientation was informative of how to map the choice (up/down) to a response (left/right). In addition, and before decision making, participants were randomly instructed by an instructed-response cue to press either the left, the right or no button (Fig. 1b). Each instructed button-press was later considered as “same” or “different” according to the last choice related button-press.

Each trial started with the instructed-response cue presented centrally for 0.25 s, followed by a fixation period of 2 s, during which participants had to respond to the instructed-response cue. Next, the choice-response mapping cue was shown centrally for 0.25 s, followed by a fixation period of 0.75 s. Then, the motion stimulus was presented for 1.5 s. The offset of the stimulus served as the go-cue to report the choice (upward/downward motion) with a button-press (left/right). The mean (across subjects) median +/− 5/95 percentile (within subject) response time was 0.63 +/− 0.37/1.42 s. Visual feedback was provided 0.1 s after the button-press by turning the fixation spot green (correct) or red (incorrect choice) for 0.1 s. During the inter-trial interval (1 s) the fixation spot was shown.

### Stimuli

Dynamic random dot patterns were presented for 1.5 s and consisted of 1500 white dots (dot diameter: 0.11 deg) on a black background, moving at 10 deg/s according to the “random direction, different rule”^17^ in a circular aperture of 7.70 deg diameter. In the upward-moving stimulus, a fraction of dots moved coherently upwards, whereas in the downward-moving stimulus, a fraction of dots moved coherently downward. In both stimuli, noise dots had a life-time of exactly 1 frame. All instructed-response cues and choice-response mapping cues had the same luminance (38 cd/m²) and size (0.31 deg diameter) as the fixation spot.

### Setup

Participants were seated in a dimly lit room in front of a screen at a distance of 58 cm. They were observing stimuli presented on a CRT monitor (Eizo Flexscan F931). The screen was controlled by a NVIDIA GeForceGTX 460 graphics controller with a refresh rate of 60 Hz at a spatial resolution of 1024 × 768 pixels. The experiment was controlled using PsychToolbox^18^ for MATLAB.

### Procedure

Before the recording, participants practiced the task for at least 10 minutes. Then, they completed a staircase procedure with 250 trials to determine a level of coherence near the individual perceptual threshold (staircase target: 66% correct choices; average motion coherence: 3.8%). The staircase was followed by two experimental sessions with 456 trials each. During the experimental sessions, the coherence level of the stimuli was further adjusted every 50 trials, to target 66% correct performance.

### Eye movement recordings

Throughout the experiment, we recorded the participants’ eye movements with an infrared-video based eye-tracker (Arrington Research Inc., USB-220). This ensured continuous fixation.

### Correlation analyses and statistics

To quantify relations between nominal behavioral variables (responses”left” or “right” and choices “up” or “down”) we used Pearson’s correlation coefficient for binary variables (Phi coefficient). To assess statistical significance of correlation coefficients at the group level, we Fisher-z-transformed subjects’ correlation coefficients and applied two-tailed t-statistics across subjects unless noted otherwise.

All analyses were performed in MATLAB (MathWorks Inc., Natick, USA) using custom software.

## Data availability

The data that support the findings of this study are available from the corresponding authors upon request.
